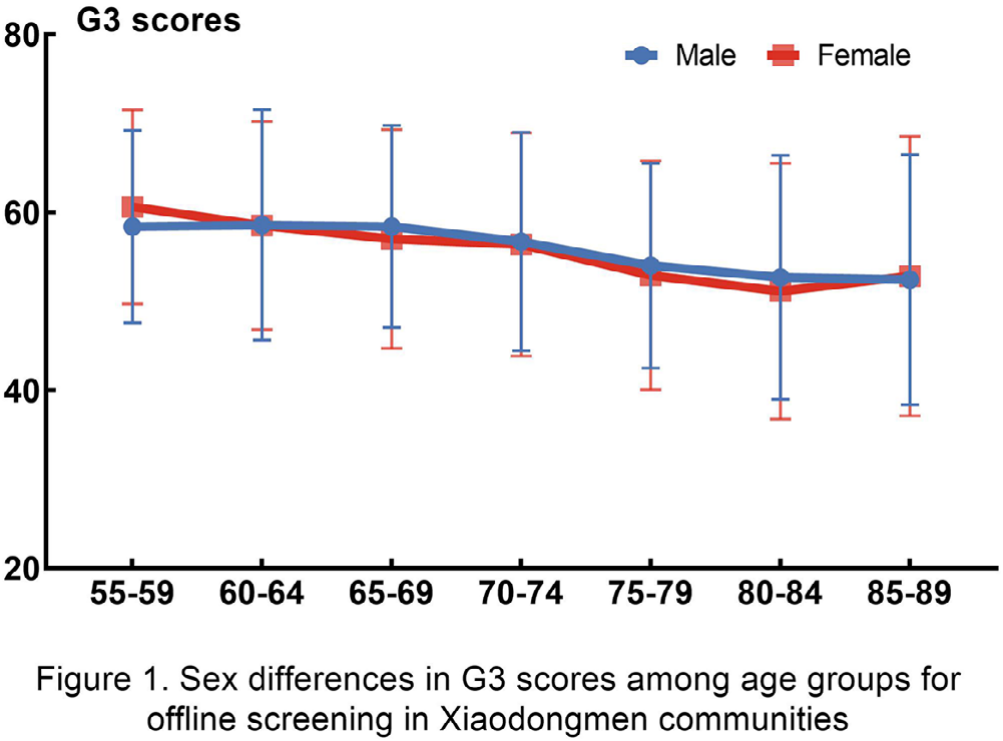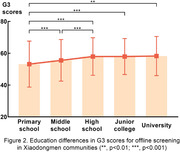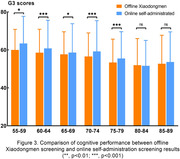# Cognitive Screening of Community‐Dwelling Older Adults in Shanghai Using a Game‐based Mobile Screening Tool: A Cross‐Sectional Study

**DOI:** 10.1002/alz.088953

**Published:** 2025-01-09

**Authors:** Jingnan Wu, Zhixing Zhou, Huanhuan Xia, Nan Chen, Yatian Li

**Affiliations:** ^1^ Shanghai Bestcovered Limited, Shanghai China

## Abstract

**Background:**

Game‐based Cognitive Assessment – 3‐minute Version (G3) is a self‐administrated mobile screening tool for detecting early cognitive impairments among Chinses community‐dwelling older adults. Released as a WeChat and Alipay mini‐programs, G3 offers a convenient and cost‐free means for individuals to evaluate their cognitive functions online. This study aimed to investigate the cognitive performance of older adults in the Shanghai community with G3. Additionally, a comparison was made between the cognitive performance of older adults who participated in the screening offline and those who proactively participated online.

**Method:**

Between June and October 2023, an offline cognitive screening with G3 tests was performed in Xiaodongmen subdistrict of Shanghai. And G3 results of 3344 participants aged 55‐89 years old were enrolled for restrictive analysis. Simultaneously, G3 online assessment records between January and December 2022 completed by 64535 individuals aged 55‐89 years old from various regions of China, were reviewed and compared to the offline screening results.

**Result:**

Among older adults aged 55 to 89 in Xiaodongmen communities, the average G3 score (mean ± stand deviation) was 56.82±12.47. Furthermore, the mean G3 score for males and females was 57.23±12.29 (n = 1569) and 56.46±12.61(n = 1775), respectively, both of which exhibited a trend of cognitive decline with increasing age (Figure 1). Moreover, older adults with a high school education or higher demonstrated significantly better cognitive performance compared to those without (p<0.05, Figure 2). When comparing the online self‐administrated G3 results of age‐matched older adults, both cohorts displayed a similar trend of cognitive decline with increasing age. However, the average G3 score obtained from Xiaodongmen subdistrict was statistically lower than that of the online assessments (p<0.05, Figure 3).

**Conclusion:**

This study characterized the cognitive function among older adults in Shanghai and enhanced the feasibility of G3 tool for large‐scale cognitive screening in Chinese communities. Nonetheless, due to the notable disparity between online and offline screening results, further investigations are warranted to explore the underlying mechanisms.